# SVcnn: an accurate deep learning-based method for detecting structural variation based on long-read data

**DOI:** 10.1186/s12859-023-05324-x

**Published:** 2023-05-23

**Authors:** Yan Zheng, Xuequn Shang

**Affiliations:** School of Computer Science, Northwestern Polytechnical University, West Youyi Road 127, Xi’an, 710072 China

**Keywords:** Long-read sequencing data, Structural variations, SV caller, Deep learning

## Abstract

**Background:**

Structural variations (SVs) refer to variations in an organism’s chromosome structure that exceed a length of 50 base pairs. They play a significant role in genetic diseases and evolutionary mechanisms. While long-read sequencing technology has led to the development of numerous SV caller methods, their performance results have been suboptimal. Researchers have observed that current SV callers often miss true SVs and generate many false SVs, especially in repetitive regions and areas with multi-allelic SVs. These errors are due to the messy alignments of long-read data, which are affected by their high error rate. Therefore, there is a need for a more accurate SV caller method.

**Result:**

We propose a new method-SVcnn, a more accurate deep learning-based method for detecting SVs by using long-read sequencing data. We run SVcnn and other SV callers in three real datasets and find that SVcnn improves the F1-score by 2–8% compared with the second-best method when the read depth is greater than 5×. More importantly, SVcnn has better performance for detecting multi-allelic SVs.

**Conclusions:**

SVcnn is an accurate deep learning-based method to detect SVs. The program is available at https://github.com/nwpuzhengyan/SVcnn.

**Supplementary Information:**

The online version contains supplementary material available at 10.1186/s12859-023-05324-x.

## Background

Structural Variations (SVs) [1] refer to large-scale mutations (with length $\ge 50$ base pairs) in a genome, which mainly includes deletions, insertions, inversions, translocations, and complex forms of multiple events. Although SVs are less frequent than SNPs and small indels, recent research has shown that they play an important role in many genetic diseases, such as cancer, autism, and Alzheimer’s disease [2–4]. Additionally, SVs have a significant impact on evolution [5, 6], gene expressions [7], and phenotype [8, 9]. Furthermore, SVs also play an essential role in plants regarding direct phenotype [10].

Over past decades, the problem of calling SVs in the whole genome has been well studied. Initial studies prioritized SV detection from short reads (100–150 bp). Various methods are developed, such as Delly [11], Lumpy [12], Pindel [13], Manta [14], Gustaf [15] and SurVIndel [16]. However, these approaches were limited by the short-read length resulting in suboptimal sensitivity. With the development of long-read sequencing technologies (such as PacBio [17] and ONT [18]), it has become feasible to detect SV with high sensitivity since the longer read length enables more accurate alignment of reads to the reference genome. Consequently, several new SV callers based on long-read data have been developed recently, including DeBreak [19], cuteSV [20], Sniffles [21], NanoSV [22], picky [23], SVIM [24], PBHoney [25] and SVision [26].

Although current long-read SV callers have made great strides, they still have some issues that can be further optimized. One issue is that an SV in highly repetitive regions may be divided into multiple smaller SVs due to incorrect alignments. As a result, many false SVs may be generated in repeat regions, causing most current long-read SV callers to miss the true SVs. This problem is particularly common to most long-read SV callers, especially on ONT data, which has a higher error rate (approximately 5–15%) than Pacbio data [27].

Besides the issue mentioned above, existing methods struggle with resolving multi-allelic SVs. Since humans have a diploid genome, reads from homologous chromosomes will be mapped to the same position when aligned to a reference genome. Consequently, different SVs may exist in this position, which is referred to as multi-allelic SVs. However, existing methods often only detect one of the SVs present in these cases.

In addition to the two issues mentioned above, we have discovered another issue with existing SV callers - the output result contains numerous false SVs. Related studies have shown that each human has about 20,000 structural variations on average [28]. However, most current SV callers output over 29,000 SVs. This means that even if an individual has around 20,000 SVs, the existing SV callers would still output several thousand false SVs. To address this issue, we consider using deep learning to filter out these false SVs. Over the past few years, there has been a significant increase in the amount of research focused on deep learning, and many researchers have employed it in SV studies. The main methods include DeepSVFilter [29], DeepCNV [30], DeepSV [31], CNV-espresso [32], and Cue [33], etc. Therefore, we are evaluating the potential of deep learning in eliminating false SVs to enhance the precision of detection results.

Based on our observations, we have developed a novel SV caller method called SVcnn. This method accurately detects DELs, INSs, DUPs, and INVs. SVcnn is a convolutional neural network (CNN) based method consisting of three parts. The first part identifies candidate SV regions from the bam file. The second part converts the candidate SV regions into images and builds the LetNet model. The third part filters false SVs through the LetNet model and outputs the final SVs. We tested SVcnn and other callers on three real datasets (CHM13, HG002, HG00733) and found that SVcnn outperforms current methods with an improved F1-score of 2–8% when the read depth is greater than 5×. Furthermore, SVcnn can identify more multi-allelic SVs with fewer false SVs.

## Results

### SVcnn has better performance than traditional methods in new benchmark

We downloaded the ONT long-read data (CHM13, HG002, and HG00733), aligned them into reference hg38, and got bam files (The Additional file 1: Table S1 contains the long-read data and reference link, while Sect. “Data sources and program commands” contains the Linux commands). Subsequently, we ran SVcnn, DeBreak (newest version), cuteSV (version=2.0.2), Sniffles2 (version=2.0), and NanoSV (newest version) on the bam files to obtain results. Additional file 1: Sect. 2 shows the detailed numbers of deletions and insertions identified by different methods and in the benchmark. To evaluate the performance of various SV callers, we also utilized the Generating benchmark method to generate new benchmarks (Additional file 1: Sect. 3). The method for verifying if an SV caller output an SV in the benchmark is available in Additional file 1: Sect. 7. The reason why we didn’t show the INV and DUP result is that we can not find a high-confidence INV and DUP benchmark at present. There is also no perfect INV and DUP caller method. Hence, we only showed the results of DEL and INS.

**Fig. 1:**
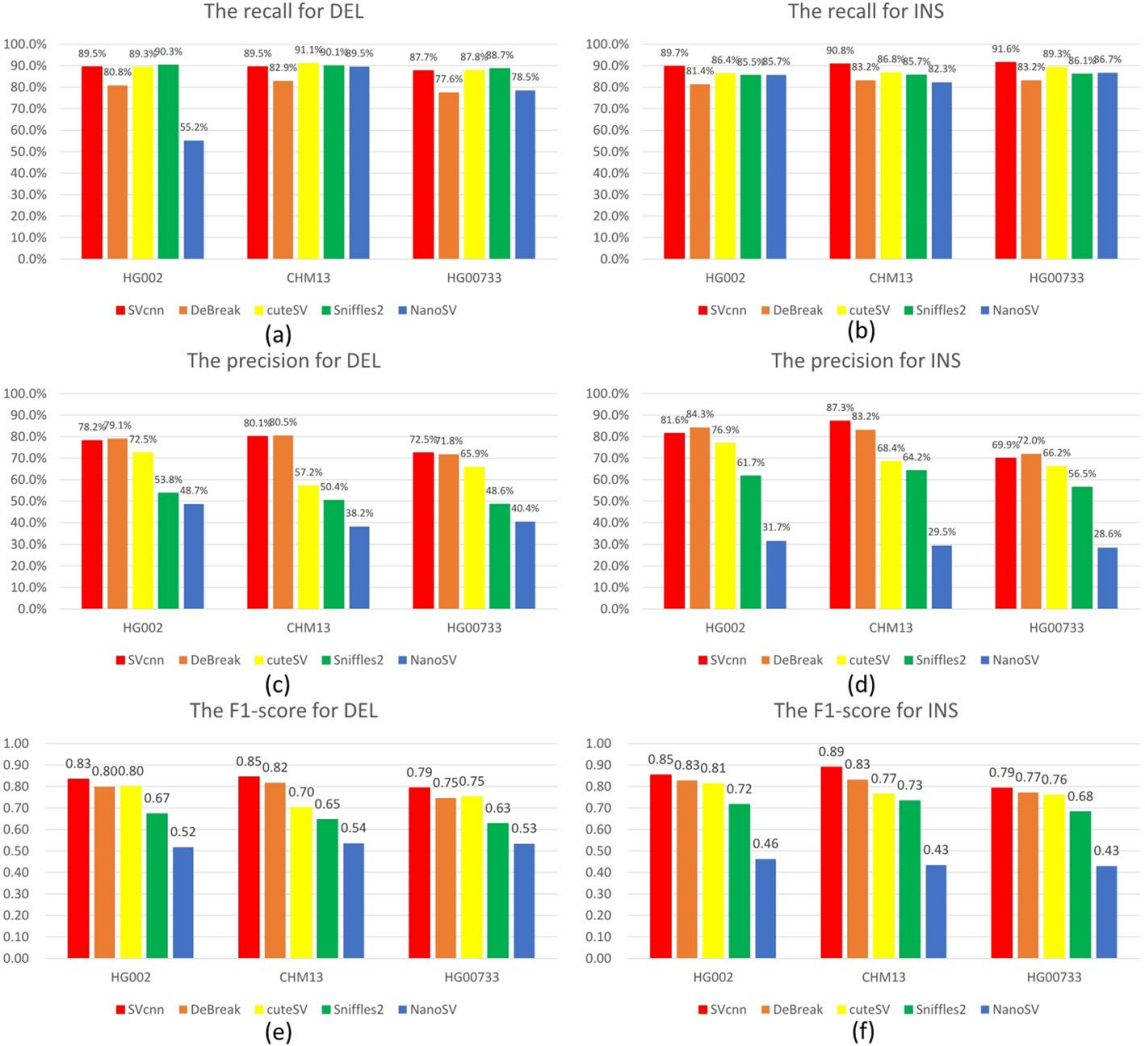
The figure shows the recall, precision, and F1-score of different SV callers using three ONT datasets for HG002, CHM13 and HG00733. **a** The recall of DELs in three datasets. **b** The recall of INSs in three datasets. **c** The precision of DELs in three datasets. **d** The precision of INSs in three datasets. **e** The F1-score of DELs in three datasets. **f** The F1-score of INSs in three datasets. From the histogram, we can clearly see that SVcnn achieved the best results in F1-score

Figure 1 shows the recall, precision, and F1-score of SVcnn, DeBreak, cuteSV, Sniffles, and NanoSV in CHM13, HG002, and HG00733. Additional file 1: Sect. 2 shows the detailed data. The figure indicates that SVcnn achieved the highest F1 score in all datasets. Moreover, the recall and precision values of SVcnn are almost identical to those of the best-performing methods. Among other callers, DeBreak is the newest and the second-best SV caller. The comparison between the SVcnn result and the DeBreak result revealed that the average improvement in the F1-score of SVcnn was about 4%. Therefore, we can conclude that SVcnn outperforms other methods.

**Fig. 2:**
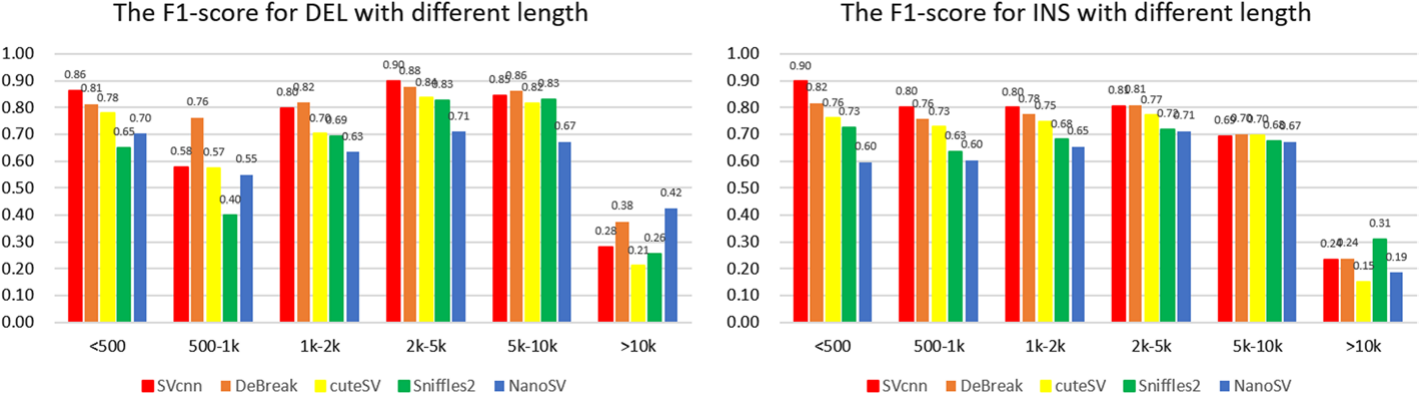
The figure shows the f1-score of different SV callers for SVs with different lengths. The histogram indicates that SVcnn has the highest f1-score when SVs are less than 500 bp. However, it performs poorly when SVs exceed 10k bp

In addition, we are interested in evaluating the performance of different SV callers in detecting SVs of various lengths. In CHM13, the SVs detected by the different methods were divided into six intervals based on length: $<500$, $500-1k$, $1k-2k$,$2k-5k$, $5k-10k$, and $>10k$. Figure 2 displays the F1-scores of the different SV calling methods across the different SV length intervals. The results show that SVcnn performs best when the SV length is less than 500 bp. And when the SV length is less than 10k bp, SVcnn performs better than most other methods on DEL and outperforms all the methods on INS. Based on different SV callers’ results, the majority of the DELs and INSs (approximately 85–90% and 78–88% respectively) have lengths less than 500 bp, and less than 1% of the SVs have lengths greater than 10k bp. Therefore, while SVcnn may perform poorly on SVs with lengths greater than 10k bp, it outperforms the other SV calling methods in the whole benchmark. Additionally, compared to other methods, SVcnn produces fewer false SVs with lengths less than 500bp (the second-best method, Debreak, exhibits similar performance). Taken together, these results explain why SVcnn and DeBreak have better precision compared to the other SV callers. Similar trends are also observed in the HG002 and HG00733 datasets.

### CNN model can improve detection performance

This section aims to show why the LetNet model can improve detection performance. To achieve this, we conducted an ablation study to evaluate the effectiveness of using the LetNet model in filtering false SVs. Firstly, we developed an SV detection program called SVnocnn, which excluded the conversion of SV regions into images and did not undergo filtration by our trained LetNet model. Subsequently, we obtained the SV results of SVnocnn on the HG002, CHM13, and HG00733 datasets. Finally, we compared these results to our benchmark, and based on the F1-scores, we were able to determine the impact of using the LetNet model in improving SV detection performance.

**Table 1:** The F1-score of SVcnn and SVnocnn

	SVcnn	SVnocnn
HG002_del	0.83	0.82
HG002_ins	0.85	0.84
CHM13_del	0.85	0.84
CHM13_ins	0.89	0.88
HG00733_del	0.79	0.77
HG00733_ins	0.79	0.78

**Fig. 3:**
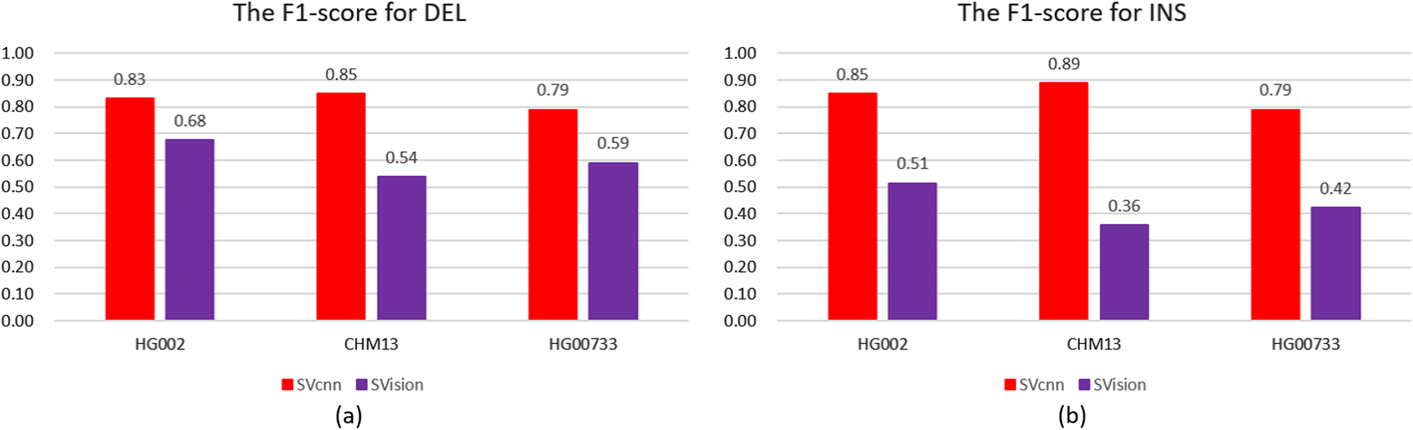
The F1-scores of SVcnn and SVision for calling **a** DELs and **b** INSs in HG002, CHM13, and HG00733. The main reason for the poor performance of SVision is that SVision outputs too many SVs (about 50,000), far exceeding the expected number (about 22,000) of SVs

According to Table 1, we have found that SVcnn’s F1-scores are superior to SVnocnn’s F1-scores across all datasets. Furthermore, regardless of the SV type, filtering the results using the LetNet model improves the performance by 1–2%. Therefore, we can conclude that using the LetNet model for filtering SV is an effective strategy. Additionally, our method outperforms traditional SV callers even without the Letnet model. Conversely, we infer that SVcnn outperforms traditional methods combined with deep learning filtering strategies. To prove this point, we compared SVcnn with deep learning-based method: SVision. The result are shown in Fig. 3.

### SVcnn has better performance than traditional methods in GIAB benchmark

**Fig. 4:**
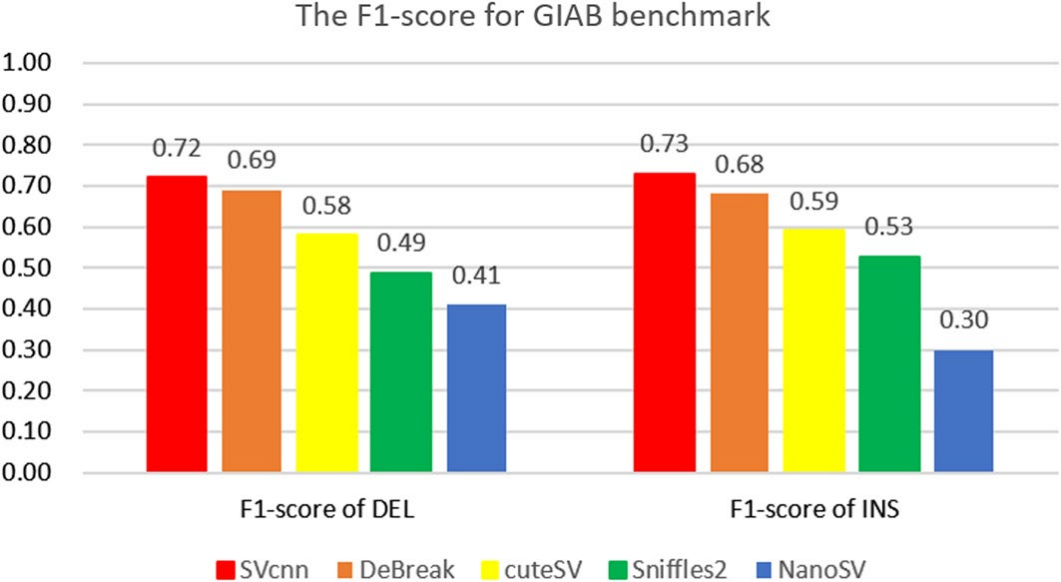
The figure shows the F1-score of different SV callers using the GIAB benchmark (HG002_SVs_Tier1_v0.6). From the histogram, we can see that SVcnn still has the best F1-score

We downloaded the HG002 ONT long-read data, aligned it into reference hg19, and obtained the bam file. Then we ran SVcnn, DeBreak, cuteSV, Sniffles2, and NanoSV in the bam file and got different results. Finally, we compared the results of the different methods with the benchmark (HG002_SVs_Tier1_v0.6) of HG002 on hg19 downloaded from the GIAB website. The F1-score for each of the four methods is displayed in Fig. 4.

From Fig. 4, we can still conclude that SVcnn has the best performance. SVcnn’s result has at least a 3% improvement over DeBreak’s. Additionally, we can see that the F1-score obtained by using the GAIB benchmark is lower compared to the F1-score in the previous section. The reason for this is that the GAIB benchmark only provides 12,742 high-confidence SVs, while the four methods all output at least 23,000 SVs. Therefore, the precision of all SV callers on this benchmark will be very low. However, many SVs that are not included in the GIAB benchmark are true SVs as related studies have shown that each human has about 20,000 structural variants on average [28]. Hence, we propose a method to generate a new benchmark. Compared with the GIAB benchmark, our new benchmark is not only more complete (contains thousands more SVs) but also contains almost all the SVs in the GIAB benchmark (see Additional file 1: Sect. 4). Therefore, we believe the results shown in Fig. 1 are more meaningful.

### SVcnn has better performance than traditional methods under different read depths

**Fig. 5:**
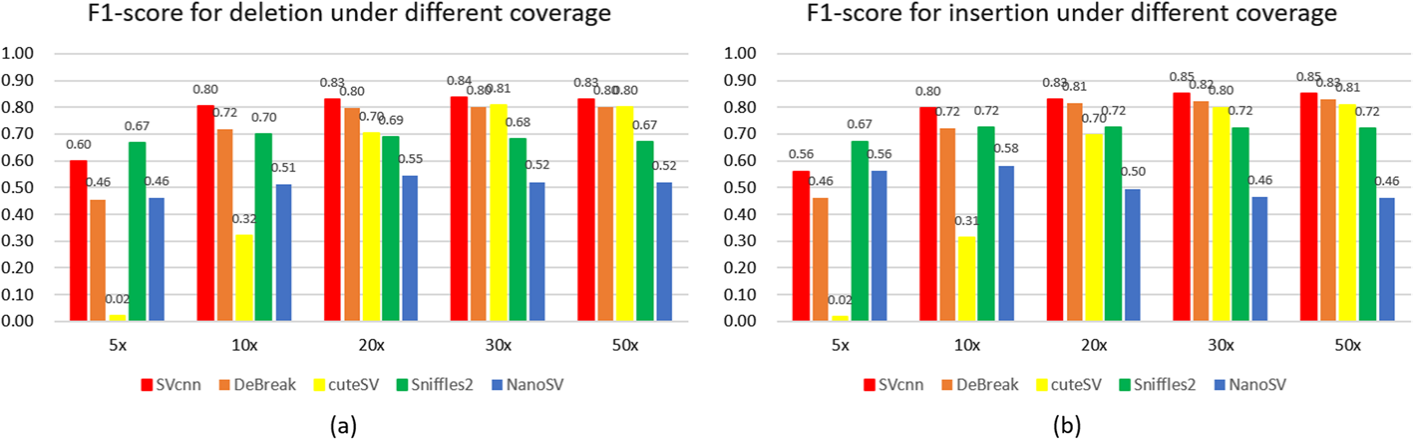
The F1-scores of SVcnn and other SV callers for calling **a** DELs and **b** INSs in HG002 under different sequencing depths. From the histograms, we can see that SVcnn has the best performance when the sequencing depth is greater than 5×. When sequencing depth is 5×, SVcnn is still the second-best method

In order to study the influence of sequencing depth, we randomly subsampled long reads from the HG002 ONT dataset at different depths: 30×, 20×, 10×, and 5×. These reads were aligned to the reference genome hg38 and produced several new BAM files. We ran five methods (SVcnn, DeBreak, cuteSV, Sniffles2, and NanoSV) on these new BAM files and evaluated their performance under different read depths. Figure 5 shows the F1-scores of these methods under different sequencing depths. From the results, it is evident that SVcnn has the highest F1-score compared to other methods when the read depth is greater than 5×. More importantly, SVcnn’s F1-score remains above 0.8 even when the sequencing depth reduces to 10×. However, Sniffles2 performs best when the read depth is 5×, and SVcnn is still the second-best method in such conditions. Moreover, the results show that the performances of Sniffles2 and NanoSV are least affected by sequencing depths, while cuteSV’s performance is most influenced by read depth.

### SVcnn has better performance for multi-allelic SVs

To test the performance of different methods for multi-allelic structural variations (SVs), we selected 243 pairs of multi-allelic deletions (DELs) (486 DELs in total) and 584 pairs of multi-allelic insertions (INSs) (1168 INSs in total) from the HG002 benchmark dataset. HG002 is a heterozygous genome, and these multi-allelic SVs (<1000bp) not only share the same type but also have a length difference of less than 20%. We then compared the results of different SV callers with these multi-allelic SVs. Table 2 presents the comparison results. The table shows that SVcnn can detect the most multi-allelic SVs (93.4% DELs and 89.6% INSs). The second-best method, DeBreak, can only identify 74.6% of multi-allelic DELs and 73.1% of multi-allelic INSs. This suggests that most current SV callers can only output one SV for a pair of multi-allelic SVs.

**Table 2:** The number of multi-allelic SVs detected by different methods for 243 pairs of multi-allelic DELs and 584 pairs of multi-allelic INSs

	SVcnn	cuteSV	Sniffles2	DeBreak	NanoSV
486 DELs	454	316	357	363	323
1168 INSs	1047	795	750	854	853

**Table 3:** The number of multi-allelic SVs detected by different methods for CHM13

	SVcnn	cuteSV	Sniffles2	DeBreak	NanoSV
DELs	122	965	1732	766	8651
INSs	227	1525	2075	1207	17,909

Next, we checked the multi-allelic SVs in CHM13. As most regions of CHM13 are homozygous, we considered it to be a homozygous genome. Therefore, there should not be any multi-allelic SVs in CHM13. If SV callers output multi-allelic SVs in CHM13, those SVs must be false positives. We counted the number of multi-allelic SVs identified by different methods in CHM13, and the results are shown in Table 3. From Table 3, it is evident that SVcnn had the fewest multi-allelic SVs, which also implies that it produced the fewest false positives. DeBreak was the second-best method, with only 766 multi-allelic DELs and 1207 multi-allelic INSs. However, NanoSV had the worst result, as it generated a large number of multi-allelic SVs (false positives). This is also the reason for the low precision value of NanoSV.

### SVcnn has better performance than methods based on deep learning

In addition to the traditional SV detection methods mentioned above, we also compared SVcnn with deep learning-based methods. We applied the latest version of SVision to the bam files of HG002, CHM13, and HG00733 to obtain results. Then we choose a more meaningful new benchmark to evaluate the performance of SVision. Figure 3 illustrates the F1-scores of SVcnn and SVision in CHM13, HG002, and HG00733.

Based on Fig. 3, we observed that SVcnn has significantly better performance than SVision. There are two primary factors that account for this difference. Firstly, SVision outputs an excessive number of SVs, particularly INS (averaging over 30,000), which is higher than the expected number in an individual. Related research has shown that the total SVs in an individual are only about 22,000. Secondly, SVision’s main purpose is to detect complex SVs, hence its capability in detecting simple SVs is limited.

### The running time of different methods

We evaluated the speed performance of several SV callers: SVcnn, vapor, cuteSV, Sniffles2, and DeBreak, on the HG002 ONT dataset with a sequencing depth of 50×. The experiments were conducted on the same Linux server with a memory capacity of 1.4T. We recorded the running time of each SV caller and provided their respective running commands in the Additional file 1: Sect. 1.4. The detailed data are presented in Table 4.

**Table 4:** The run time of different methods

	SVcnn	cuteSV	Sniffles2	DeBreak	NanoSV
Time	15 h 42 m	16 m	20 m	48 m	5 d 40 m

Based on Table 4, we have observed that SVcnn’s processing speed is slow, with its performance being only better than NanoSV. This can be attributed to the fact that a significant amount of time is spent converting SV-containing regions into images. On the other hand, cuteSV has the fastest processing speed due to being a C language program.

## Discussion

From the results section, we can conclude that our method SVcnn outperforms existing traditional methods, including DeBreak, cuteSV, Sniffles2, and others. In addition, we conducted an ablation experiment in the Results section to demonstrate the effectiveness of deep learning. The experimental results showed that the deep learning filtering strategy only improved the results by 1–2%, while SVCNN’s improvement was 2–8%. This indicates that even without the deep learning filtering strategy, our method still outperforms traditional methods. Conversely, we infer that SVcnn outperforms traditional methods combined with deep learning filtering strategies.

In addition to traditional methods, there are also some SV callers that use deep learning to detect SV or explore the internal structure of SV, such as SVision [26]. We also ran SVision and compared its results with SVcnn, and found that SVcnn still outperformed SVision. The main purpose of SVision is to explore the internal structure of complex SVs, so its performance is not as good as SVcnn in the overall benchmark. Therefore, we can conclude that SVcnn is more accurate than existing SV callers.

## Conclusions

In past research, numerous long-read based SV calling methods have been developed. Generally, long-read based SV callers outperform short-read ones as the former has a more reliable alignment. However, we have observed that current SV callers face certain challenges while dealing with SVs in repeat regions and multi-allelic SVs. Therefore, we propose a new method, called SVcnn, which uses long-read data to detect SVs. The innovation of this method lies in the use of a LetNet model for filtering SVs. Compared to existing methods, SVcnn offers the following advantages:

1. SVcnn has the best F1-score for all datasets.
2. SVcnn can filter more false SVs.
3. SVcnn still has good performance when the sequencing depth is low.
4. SVcnn has better performance for multi-allelic SVs.

There are still some limitations to SVcnn. For complex SVs (where two different types of SVs are adjacent), SVcnn still does not perform well. Additionally, for BNDs (a single type of SV), SVcnn is still unable to detect them. Furthermore, the speed of SVcnn is slow because it needs to convert the regions where the SVs are located into images. In the future, we intend to further study the detection of BNDs and complex SVs, as well as optimize the speed of SVcnn.

## Methods

### Details of other SV callers and testing data

Almost all SV callers are developed based on short-read or long-read data. In the previous section, we stated that SV callers based on long-read data have higher sensitivity than those based on short-read data. Therefore, our method is also developed based on long-read data. The two most popular long-read technologies are PacBio and Oxford Nanopore (ONT). Compared to PacBio data, the reads in ONT data have a higher error rate. Therefore, it is more challenging to detect SVs on ONT data. Hence, we chose to test our performance on ONT data in this paper.

There are so many long-read SV callers, and testing them individually was impractical. Therefore, we selected four traditional methods from many long-read SV callers: NanoSV [22], Sniffles2 [34],cuteSV [20] and DeBreak [19]. These four methods are selected for their high accuracy in detecting SVs. NanoSV [22] is the only method that targets ONT data to call SVs. Sniffles2 [34] is the second version of the most popular SV caller. CuteSV [20] is the method that has been shown to have the highest recall value. DeBreak [19] is the newest method, which was published in Nature Communications. In addition to the traditional search methods, we select an SV caller based on deep learning: SVision [26]. SVision is a method published in Nature Methods in 2022, which uses a deep learning model to explore the internal structure of complex SVs.

To test the performance of SV callers, we downloaded the HG002 benchmark (HG002_SVs_Tier1_v0.6) from GIAB, which is available at https://ftp-trace.ncbi.nlm.nih.gov/giab/ftp/data/AshkenazimTrio/analysis/NIST_SVs_Integration_v0.6/HG002_SVs_Tier1_v0.6.vcf.gz. This benchmark is widely accepted by researchers in related fields as the most reliable benchmark. We selected only the pass-type SVs, which have very high confidence, resulting in a total of 5463 DELs and 7279 INSs. These 12,742 SVs have very high quality, and their SV lengths and breakpoints are correct. Therefore, we consider these SVs to be 100% accurate. We aligned the long-read sequencing data to the GRCh37 reference genome and applied SVcnn and other methods in the alignment file. Finally, we compared the results obtained from different methods.

Except for this benchmark, finding such a high-quality benchmark in other datasets is hard. Additionally, although this benchmark (HG002_SVs_Tier1_v0.6) is of high quality, it is not comprehensive. Related studies have shown that each human individual has an average of 20,000 structural variants [28], while this benchmark only includes 12,742 SVs. This means that the benchmark is likely to miss thousands of true SVs. Besides this, using just one benchmark to test performance may not be sufficient.

To resolve this problem, we utilized results from different methods on HiFi reads [35] and genome assemblies of the sample (just like HG002) to further enhance the benchmarks. HiFi reads are generated using circular consensus sequencing (CCS) mode on PacBio long-read systems, with an impressively low error rate of only about 0.1%. This data can be used to obtain a much-improved benchmark. The detailed steps to create this new benchmark are outlined in Additional file 1: Sect. 3.

Finally, we selected three sample genomes (CHM13, HG002, and HG00733) and downloaded their genome assemblies to create a new benchmark. The download links for these sample genome assemblies are provided in Additional file 1: Table S2. These three new benchmarks were used along with HG002_SVs_Tier1_v0.6 to test the performance of different SV callers.

### Overview of SVcnn

According to our observations, we have found that the current methods perform poorly in detecting multi-allelic SVs and SVs in repeat regions. Therefore, we propose a new method called SVcnn to detect SVs accurately throughout the whole genome. Utilizing long-read sequencing data, SVcnn is an accurate SV caller based on deep-learning models. Compared with existing SV callers, SVcnn can overcome some challenges that are not solvable by other methods. For example, SVcnn uses hierarchical clustering to identify if a region contains multi-allelic SVs. Moreover, SVcnn utilizes the LetNet model to distinguish whether an SV is a true SV or not. Hence, SVcnn outperforms other SV callers in terms of its precision and accuracy.

**Fig. 6:**
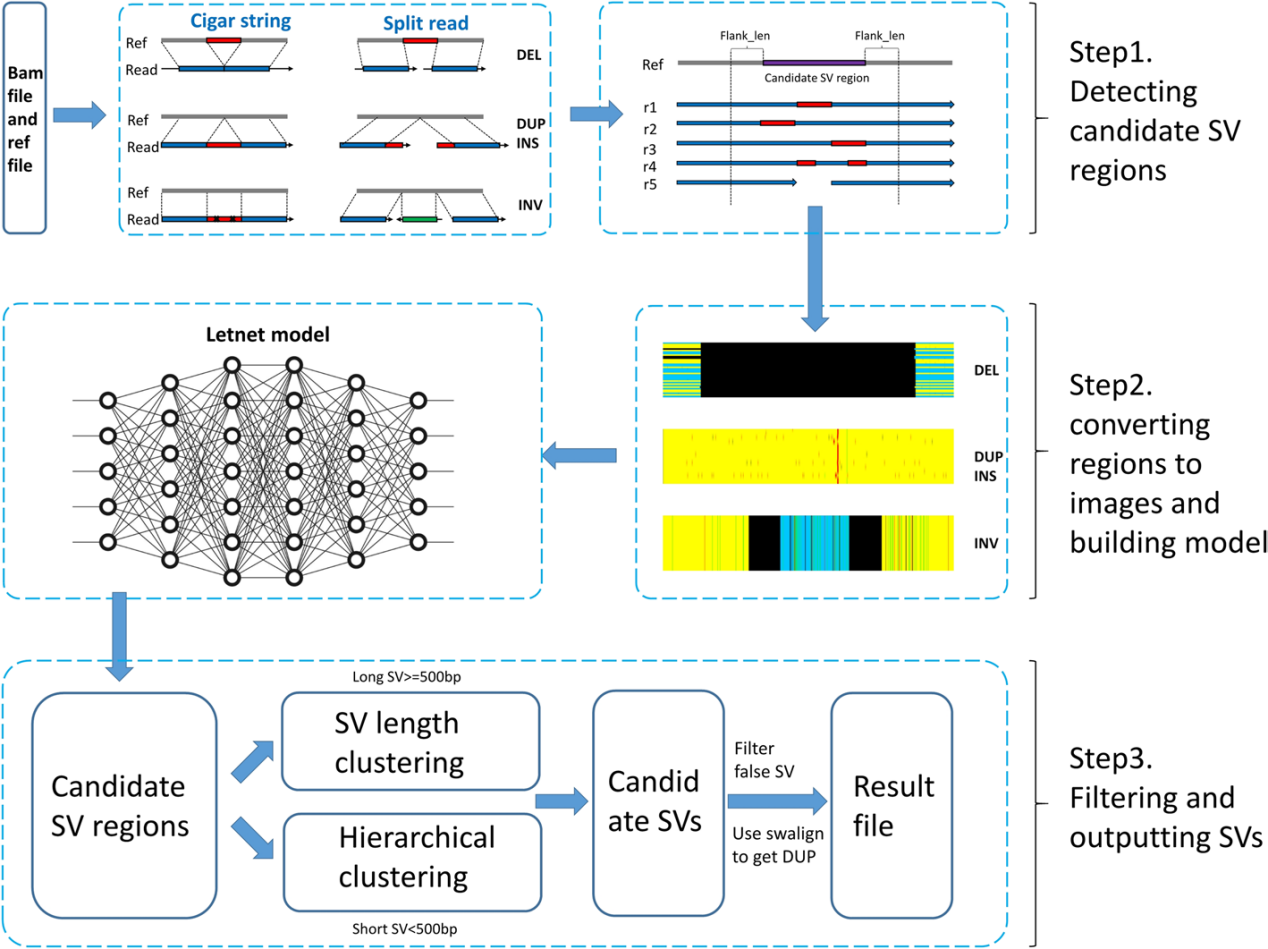
The overview of SVcnn. There are three main steps in SVcnn. (1) Detecting candidate SV regions, (2) Converting regions to images and building model, (3) Filtrating and outputting SVs

The input of SVcnn consists of (i) a sorted long read bam file and (ii) a reference file. SVcnn mainly consists of three main steps: (1) Detecting candidate SVs, (2) Converting to image and building model, (3) Filtering and outputting SVs. Figure 6 illustrates the steps involved in SVcnn, while the detailed steps are explained in the following sections.

#### Detecting candidate SV regions

The first step of SVcnn is to detect candidate SV regions (regions where SVs may exist). Before running the method, we need to download the long-read sequencing data and the reference genome. Next, we should align the long reads into the reference genome using current aligner methods and obtain the SAM file. Finally, we should convert the SAM file into a sorted BAM file using samtools [36]. The sorted BAM file and reference genome will be processed as input data by SVcnn.

#### Estimating the parameters

Before detecting the SVs, we process the bam file and estimate the parameters. We randomly select 1000 nodes from the reference and calculate the average coverage of these nodes using the following formula:

$$\begin{aligned} average\_coverage_{bamfile}=\frac{1}{1000}\sum _{i=1}^{1000} coverage_{i} \end{aligned}$$

In the genome, there are some regions that have very high coverage, sometimes dozens of times higher than the average coverage. This indicates that reads from other regions align to this region, and the similarity between these regions is very high. For these regions, it becomes difficult to determine which reads belong to which specific region. Additionally, when we convert SVs into images, analyzing these regions would take up a lot of time. Therefore, we have decided to discard these high-coverage regions. If a region has a coverage exceeding five times the average, we will filter out the SVs in that region.

#### Identifying candidate SV regions

From the aforementioned steps, we obtained a sorted BAM file using aligner methods such as minimap2 [37] and NGMLR [21], and samtools. SVcnn scans the entire sorted BAM file to extract all candidate SV regions. A region may contain two different SVs due to the existence of multi-allelic SVs. Therefore, we first identify the regions where SVs may exist before identifying the exact SVs. Two main methods can be used to detect candidate SV regions. The first method involves detecting candidate SV regions by checking the CIGAR string, while the second method uses split reads (as shown in step 1 of Fig. 6). Please note that duplications cannot be directly identified from the bam file. Duplications can be considered a special type of insertion, which shares the same features as an insertion in the bam file. Therefore, we first identify all insertions and then compare their insertion sequences with the reference to determine whether the SV is an insertion or duplication.

1. **Detect candidate SV by CIGAR strings.** To detect candidate SVs using CIGAR strings, we scan the sorted BAM file and check the alignments one by one. To reduce false positives in the results, we only retain alignments with a MAPQ greater than 20 (by default). For each retained alignment, we examine the CIGAR string and record all insertions (I) and deletions (D) longer than 30bp as candidate SVs.
2. **Detect candidate SV by split read.** For large structural variants (SVs), it can be challenging to identify SV signatures directly from the cigar string. This is because reads near these large SVs tend to split into multiple alignments, generating split reads (similar to the middle part of step 1 shown in Fig. 6). Therefore, we inspect the sorted BAM file and retain primary alignments (MAPQ >20) that are divided into multiple parts. Next, SVcnn identifies candidate SVs using the following criteria. More detailed steps are outlined in Additional file 1: Sect. 5.
  1. Suppose the read has two alignments and the two alignments are on the same chromosome and on the same strand. This split alignment probably contains a DEL or INS. We respectively record the distance of two alignments on reference and read (ref_distance, read_distance). Next, we get the SV by calculating the difference of read_distance and ref_distance.
  2. Suppose the read has two alignments or three alignments and these alignments are on the same chromosome and have different strands. This split alignment probably contains an INV. We record the positions of the breakpoints and get the INV.

From the above two steps, we can obtain detailed information about each candidate SV. We use a length-6 tuple to record every candidate SV, which consists of *(chr_name, s_pos, e_pos, length, type, read_name)*. Here, chr_name refers to the chromosome name; s_pos and e_pos represent the start and end positions of the SV respectively; length denotes the SV length; type indicates the type of SV (e.g., DEL, INS, INV); and read_name specifies the name of the read where this SV is located.

After obtaining the tuple for each candidate SV, we have to merge the tuples that represent the same SV in order to generate candidate SV regions. Since we record the SV in the form of a tuple for each read, if an SV has N supporting reads, it will be recorded N times. The method used to combine the candidate SVs into candidate SV regions is described in the following paragraph.

For each tuple generated in the previous step, we extract a length-5 tuple (chr_name, s_pos, e_pos, type,1). This new tuple is recorded as the initial region, where the final value ’1’ represents the support read number. Let region1=(chr_name1, s_pos1, e_pos1, type1,1) and region2=(chr_name2, s_pos2, e_pos2, type2,1) be two initial regions. If these two regions meet the following criteria, we merge them into one region.

1. chr_name1=chr_name2.
2. type1=type2.
3. abs(max(s_pos1,s_pos2)-min(e_pos1,e_pos2)) $\le$ 1000.

The third criterion means the distance between the two regions is less than 1000bp. After merging the two regions into a new region, we record the new region as (chr_name1, min(s_pos1,s_pos2), max(e_pos1,e_pos2),type1,2). Because the regions on the two reads are merged together, the last value changes from 1 to 2. We repeat this step until the remaining regions cannot be merged, and finally, we get a list of candidate SV regions. To remove the effects of noise, we only retain candidate SV regions with more than 3 supporting reads (the last value of the tuple).

#### Converting regions to images and building model

This section is mainly divided into two parts: the first part involves converting the region into an image, while the second part focuses on training the LeNet model.

#### Converting regions to images

From the steps outlined above, a list of candidate SV regions is obtained. In this subsequent step, we intend to convert these regions into images. To obtain more comprehensive information on SVs, a flank_len is set for each candidate region, with its value being the minimum of (200, 2*region_len). Each candidate region is then converted into an image.

We use a five-color image to represent each candidate region. For a region described by (chr_name, s_pos, e_pos, type, support_read_num), all alignments near the region are checked and each character of the corresponding CIGAR string is converted into a pixel. The alignments of each read occupy a row in the image. The following rules govern the conversion process (refer to step 2 of Fig. 6 for examples of DEL, INS, and INV):

1. The Match of alignment with a plus strand (+) is represented as a yellow pixel.
2. The Match of alignment with a minus strand (−) is represented as a blue pixel.
3. The DEL of alignment is represented as a black pixel.
4. The INS of alignment is represented as a red pixel.
5. The X (mismatch) of alignment is represented as a green pixel.

In the image, each line represents a read, and each column represents a position on the reference. For each candidate SV region (chr_name, s_pos, e_pos, type,support_read_num), the converted image starts at position (s_pos-flank_len) and ends at position (e_pos+flank_len). However, there is a problem in this case: INS does not occupy a position on the reference. This means that if we use columns to represent the position on the reference, the INS will not be displayed in the image. As a result, such images cannot display INS information. To solve this problem, we implemented the following method. In the bam file, M (match) occupies most of the positions, so we can replace part of M with I (INS) according to the length of the INS. In this paper, for every 10-bp INS, we replaced an M on the reference with an I. For example, if the length of an INS is 50 bp, we find 5 Ms on the reference near the INS and replace them with 5 Is. After the replacement, we convert this region into an image, and a red vertical line representing the INS can be seen in the image. A specific example can be seen in Step 2 of Fig. 6.

#### Building LetNet model

From the previous step, we have obtained images that are converted from candidate SV regions. In this step, we will be training a Convolutional Neural Network (CNN) model, which is why our method is called SVcnn. The CNN model we have chosen is the LeNet model [38]. The LeNet model was one of the first convolutional neural networks developed by Yann LeCun et al and contributed to the advancement of deep learning. Since its inception in 1988, after years of research and multiple successful iterations, this pioneering work has been named LeNet. The LeNet model comprises three convolutional layers, two subsampling layers, and two fully connected layers. The detailed parameters of the seven layers are shown below:

1. The first layer C1 is a convolutional layer and the input data is 224 * 224. The layer has six convolution kernels of 5 × 5 and the size of feature mapping is 220 * 220.
2. The second layer S2 is the subsampling layer that outputs 6 feature graphs of size 110 * 110. Each cell in each feature map is connected to 2 * 2 neighborhoods in the corresponding feature map in C1.
3. The third layer C3 is a convolution layer with sixteen convolution kernels of 5 * 5. the output size of C3 is 106 * 106.
4. The fourth layer S4 is similar to S2, with size of 2 * 2 and output of sixteen 53 × 53 feature graphs.
5. The fifth layer C5 is a convolution layer with 120 convolution kernels of size 5 * 5. The output size of feature mapping is 49 * 49.
6. The sixth layer F6 is fully connected to C5, and 84 feature graphs are output.
7. The seventh layer F7 is also a fully connected layer and 4 feature graphs are output (The 4 feature graphs represent DEL, INS, INV, and noSV).

Because the LetNet model requires a fixed input image size, it is necessary to normalize previously obtained images. This is achieved by utilizing the resize function in the Python library which resizes the image to a uniform size of 224Ã–224 pixels. Subsequently, the training dataset becomes an essential component required for training the LetNet model. HG002_SVs_Tier1_v0.6 is currently the widely accepted benchmark dataset utilized by researchers. Therefore, we use the SVs in this benchmark as training data. However, this benchmark only includes two SVs, DEL and INS. To achieve better results, we have added INVs and noSVs to the training data. To get high-quality INVs, we find 120 common INVs from other SV callers’ results and label them as INV. In addition, we also find 7138 regions without SVs in the reference and label them as noSV. In this way, we get 20,000 training regions containing four types (5463 DELs, 7279 INSs, 120 INVs, and 7138 noSVs). Then we convert all these regions into images. Finally, we got 20,000 images as the training dataset. For the training, we shuffle the 20,000 images and apply the ten-fold cross-validation for model training. The training images are divided into 10 groups, each containing 2000 images. One of the groups is selected as the validation set during model training, leading to 10 models (ID from 0 to 9). Every trained model is applied to the validation set, and the loss and accuracy of every model are calculated. The training iterations are 5000. Ultimately, we retain the model with the highest accuracy.

After obtaining the trained LeNet model, we input the candidate SV region images obtained in the previous steps into the model. We use the model to determine the probability of an SV occurring in this region and identify the type of SV. We select the label with the highest probability as the judgment result. If the model determines that there is no SV in this region, then we discard the candidate SV region. However, if the model identifies a DEL, INS, or INV, we will calculate the exact length and breakpoints of the SV in the next section.

#### Filtrating and outputting SVs

In the previous steps, we determined whether there is an SV in a candidate SV region, as well as its type. In this section, we need to further assess whether the region has multi-allelic SVs and determine the exact length of the SVs. This section consists of two main steps. The first step involves using hierarchical clustering to identify multi-allelic SVs within a region, while the second step involves outputting the correct SVs.

#### Judging whether there are multi-allelic SVs

**Fig. 7:**
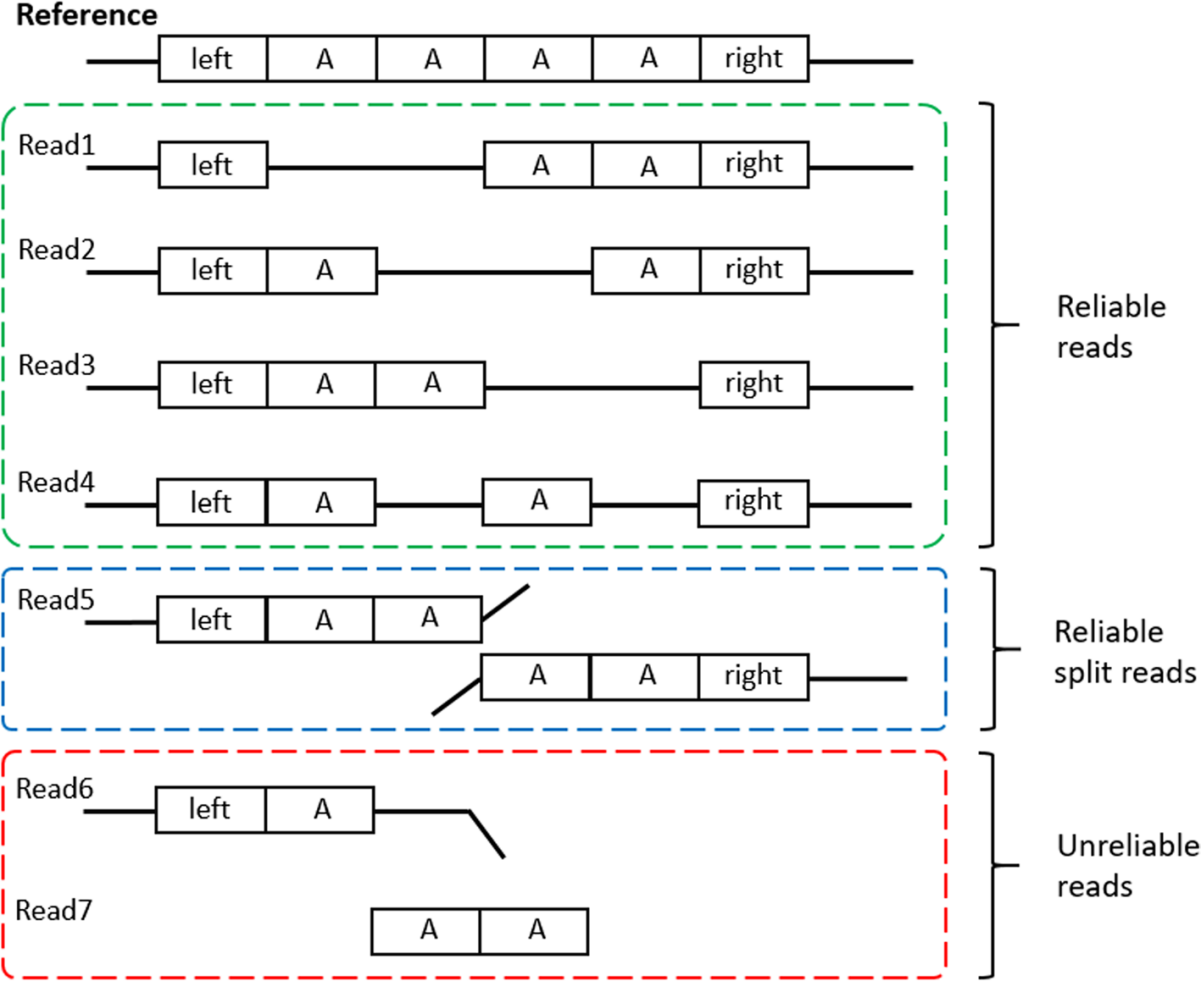
In this figure, we show the alignments of 7 different reads. The reads in the green and blue boxes will be retained as reliable reads. The reads in the red box will be filtered as unreliable reads

For each candidate SV region (after the filtration of the letnet model), we extract all reliably aligned reads and refine them to obtain a candidate SV. In candidate SV regions (especially in the repeat regions)(chr_name, s_pos, e_pos, type,support_read_num), some reads may have wrong alignments. For example, we have shown some read alignments of repeat regions in Fig. 7. The alignments of *Read*4 in Fig. 7 will give the two wrong SVs and affect the detection results. To avoid wrong alignments, we only retain two types of long reads. First, we retain long reads which can go through the region (chr_name, s_pos-1000, e_pos+1000), just like $Read1 - Read4$. Second, we retain the long reads that have two or over two alignments, just like *Read*5. As shown in Fig. 7, the reads ($Read1 - Read5$) in the green and blue boxes will be retained as reliable reads and the reads ($Read6 - Read7$) in the red box are filtrated as unreliable reads. By removing unreliable reads, SVcnn can get better results in repeat regions.

For reliable reads after the last step, if the read contains more than one candidate SVs of the same type, we will merge them. For example, for the *Read*4 in Fig. 7, it has two small wrong DELs and we try to merge them into one correct DEL. Suppose one read has two candidate SVs of type *t* like SV1 (chr_name, s_pos1, e_pos1, length1, *t*, read_name) and SV2 (chr_name, s_pos2, e_pos2, length2, *t*, read_name). We merge them as SV_m: (chr_name, min(s_pos1,s_pos2), max(e_pos1,e_pos2), length1 + length2, *t*, read_name).

Finally, we will cluster the SVs for each candidate SV region. For a candidate SV region named A with N reads, we will first calculate the average SV length by the following formula:

$$\begin{aligned} average\_SV\_len_{region A}=\frac{1}{N}\sum _{i=1}^{N} SV\_len_{i} \end{aligned}$$

After obtaining the average length, we divide the candidate SV regions into two types (long SVs and short SVs) based on whether the average SV length is greater than 500 bp.

1. **Long SVs (greater than 500bp):** First, we sort the SVs in the candidate SV region by their lengths. Each SV is recorded as a cluster, with the average length of all SVs in the cluster set as the cluster_length. If the difference in cluster_length between two clusters is less than 20%, we merge the two clusters into one. We repeat this process for the remaining clusters until no more clusters can be merged.
2. **Short SVs (less than or equal to 500bp):** For short SVs, sequencing noise has a great effect on the SV length. Therefore, when two SVs are of similar length and are short, it is difficult to distinguish between them. To address this issue, we use hierarchical clustering to separate these reads. If we observe a bimodal distribution for all SV lengths, we report two heterozygous SVs. The detailed steps are provided in Additional file 1: Sect. 8.

For every cluster (cluster_type, cluster_length) obtained, we select an SV in the cluster that is the closest to the cluster_length to represent the cluster. These selected SVs are recorded as candidate SVs and will be further processed in the next step.

#### Filtering false SVs and outputting final result

In the previous step, we obtained a set of candidate SVs. We filter out candidate SVs with lengths less than 30 bp, as they are not considered SVs. Based on our observations, there are many false SVs in simple repeat regions due to the noisy ONT data. Therefore, we use a stricter criterion to remove false SVs in simple repeat regions. Specifically, if a candidate SV is located in a simple repeat region (the method to determine if a region is a simple repeat region is described in Additional file 1: Sect. 9), we check the SV lengths of all reads in that location. If more than half of the reads have SV lengths greater than 40bp, the candidate SV will be retained.

Finally, for every retained candidate SV, we calculate the local_coverage and the support_rate. The local_coverage is the sum of reads in all clusters within this candidate SV region. The support_rate of a candidate SV is defined as follows:

$$\begin{aligned} support\_rate = cluster\_read\_num/local\_coverage \end{aligned}$$

Here, the cluster_read_num is the number of reads in the cluster representing the candidate SV. If the support_rate is greater than 20% and the number of supporting reads is greater than 3 (by default), we consider these SVs as true SVs and output them.

In the previous section, we mentioned that it is hard to directly identify DUP from the bam file because DUP shares the same features as INS. We have now obtained all the INS and will proceed to extract DUP from the INS. Firstly, we identify the insertion sequence and label it as INS_seq. Next, we extract a sequence (ins_bp-3*ins_len,ins_bp+3*ins_len) from the reference and label it as ref_seq. We then use the swalign library available in Python to align the INS_seq into ref_seq, with a match score of 2 and a mismatch score of -1. Finally, we check the alignment result to determine whether more than 80% of the INS_seq can be matched to the ref_seq. If this criterion is met, we output the SV as a DUP; otherwise, we output the SV as an INS.

### Performance measure

To evaluate the performance of different SV callers, we use three measurements: Recall, Precision, and F1-score. The F1-score is the harmonic mean of precision and recall. All three measurements are in the range between 0 and 1, and they are defined as follows:

$$\begin{aligned}&Recall:\frac{{TP}}{{benchmark\_count}} \\&Precision:\frac{{TP}}{{method\_count}} \\&F1 - score:\frac{{2*recall*precision}}{{recall + precision}} \\ \end{aligned}$$

Note that TP is the number of SVs detected by a method that appears in the benchmark, benchmark_count is the total number of benchmark SVs, and method_count is the total number of SVs predicted by the method.

## Supplementary Information

**Additional file 1**. Supplementary material.

## Data Availability

The SVcnn is available at https://github.com/nwpuzhengyan/SVcnn. Other datasets’ download links are shown in the Additional file.
