# Supplementary material for: SVcnn: an accurate deep learning-based method for detecting structural variation based on long-read data
Source: BMC Bioinformatics. 2023 May 23;24:213. doi: 10.1186/s12859-023-05324-x (PMC10207598; doi:10.1186/s12859-023-05324-x)
Supplement: Supplementary file 1 — Additional file 1. Supplementary material. [file 12859_2023_5324_MOESM1_ESM.pdf]

SVcnn: an accurate deep learning-based method for detecting structural variation based on long-read data

Yan Zheng and Xuequn Shang

May 4, 2023

1 Data sources and program commands

1.1 Data sources

The long-read download links are shown in Table S1. The repeat region of Hg19 and Hg38 was downloaded from http://genome.ucsc.edu/cgi-bin/hgTables. We choose parameter simple repeat and output in bed file format. The reference genome links and reference download links are shown in Table S1.

1.2 Commands for generating the bam files

The below commands are used to generate bam files from fastq files of HG002, CHM13, and HG00733.

minimap2 -ax map-ont –MD hg19.fa hg002_ont.fastq.gz >hg002_hg19_ont.sam
samtools view -bS hg002_hg19_ont.sam >hg002_hg19_ont.bam

minimap2 -ax map-pb –MD hg19.fa hg002_pacbio.fastq.gz >hg002_hg19_pacbio.sam
samtools view -bS hg002_hg19_pacbio.sam >hg002_hg19_pacbip.bam

minimap2 -ax map-ont –MD hg38.fa chm13_ont.fastq.gz >chm13_hg38_ont.sam
samtools view -bS chm13_hg38_ont.sam >chm13_hg38_ont.bam

minimap2 -ax map-pb –MD hg38.fa chm13_pacbio.fastq.gz >chm13_hg38_pacbio.sam
samtools view -bS chm13_hg38_pacbio.sam >chm13_hg38_pacbio.bam

minimap2 -ax map-ont –MD hg38.fa hg002_ont.fastq.gz >hg002_hg38_ont.sam
samtools view -bS hg002_hg38_ont.sam >hg002_hg38_ont.bam

minimap2 -ax map-pb –MD hg38.fa hg002_pacbio.fastq.gz >hg002_hg38_pacbio.sam
samtools view -bS hg002_hg38_pacbio.sam >hg002_hg38_pacbio.bam

minimap2 -ax map-ont –MD hg38.fa hg00733_ont.fastq.gz >hg00733_hg38_ont.sam
samtools view -bS hg00733_hg38_ont.sam >hg00733_hg38_ont.bam

minimap2 -ax map-pb –MD hg38.fa hg00733_pacbio.fastq.gz >hg00733_hg38_pacbio.sam
samtools view -bS hg00733_hg38_pacbio.sam >hg00733_hg38_pacbio.bam

1.3 Commands for Assemblytics

The below commands are used to generate the list of SVs from the reference genomes.

Table S1: The download link of different datasets

datasets	coverage	link
HG00733 ONT	72x	https://s3-us-west-2.amazonaws.com/human-pangenomics/index.html?prefix=NHGRI_UCSC_panel/HG00733/nanopore/Guppy_4.2.2
HG002 ONT	50x	https://nist-midas.s3.amazonaws.com/pdrsrv/mds2-2336/input_fastqs/HG002_GM24385_1_2_3_Guppy_3.6.0_prom.fastq.gz
CHM13 ONT	107x	https://s3-us-west-2.amazonaws.com/human-pangenomics/T2T/CHM13/nanopore/rel3/rel3.fastq.gz
HG00733 HiFi	10x	https://sra-pub-src-1.s3.amazonaws.com/ERR3822935/HG00733_hifi_r54329U_20190827_172128_1_A01.bam.1
HG002 HiFi	37x	https://nist-midas.s3.amazonaws.com/pdrsrv/mds2-2336/input_fastqs/HG002_35x_PacBio_14kb-15kb.fastq.gz
CHM13 HiFi	34x	https://s3-us-west-2.amazonaws.com/human-pangenomics/T2T/CHM13/pacbio/hifi_20kb/m64062_190806_063919.subreads.bam

Table S2: The link of different reference genome

datasets	link
HG00733 ref1	http://ftp.1000genomes.ebi.ac.uk/vol1/ftp/data_collections/HGSVC2/working/20200417_Marschall-Eichler_NBT_hap-assm/HG00733_hgsvc_pbsq2-ccs_1000-pereg.h1-un.racon-p2.fasta
HG00733 ref2	http://ftp.1000genomes.ebi.ac.uk/vol1/ftp/data_collections/HGSVC2/working/20200417_Marschall-Eichler_NBT_hap-assm/HG00733_hgsvc_pbsq2-ccs_1000-pereg.h2-un.racon-p2.fasta
HG002 ref1	https://www.ncbi.nlm.nih.gov/projects/r_gencoll/ftp_service/nph-gc-ftp-service.cgi/?HistoryId=MCID_632095d57e15fe521d4f01c4&QueryKey=1&ReleaseType=GenBank&FileType=GENOME_FASTA&Flat=true
HG002 ref2	https://www.ncbi.nlm.nih.gov/projects/r_gencoll/ftp_service/nph-gc-ftp-service.cgi/?HistoryId=MCID_6322913c0d258c17544a4ad8&QueryKey=2&ReleaseType=GenBank&FileType=GENOME_FASTA&Flat=true
chm13 ref	https://s3-us-west-2.amazonaws.com/human-pangenomics/T2T/CHM13/assemblies/analysis_set/chm13v2.0.fa.gz
hg19 ref	http://hgdownload.cse.ucsc.edu/goldenPath/hg19/bigZips/hg19.fa.gz
hg38 ref	https://hgdownload.soe.ucsc.edu/goldenPath/hg38/bigZips/hg38.fa.gz

./nucmer -maxmatch -l 100 -c 500 hg38.fa hg00733.fa -prefix hg00733_OUT
./nucmer -maxmatch -l 100 -c 500 hg38.fa hg002.fa -prefix hg002_OUT
./nucmer -maxmatch -l 100 -c 500 hg38.fa chm13.fa -prefix chm13_OUT

./Assemblytics hg00733_OUT.delta ⟨*hg00733_output*⟩
./Assemblytics hg002_OUT.delta ⟨*hg002_output*⟩
./Assemblytics chm13_OUT.delta ⟨*chm13_output*⟩

1.4 Commands for different SV callers

We run different SV callers using the following commands.
./SVcnn ⟨*input_bam*⟩ ⟨*input_reference*⟩
./debreak –bam ⟨*input_bam*⟩ -o ⟨*output_file*⟩
./sniffles2 -m ⟨*input_bam*⟩ -v ⟨*output_file*⟩
./cuteSV ⟨*input_bam*⟩ ⟨*reference*⟩ ⟨*output_file*⟩ ⟨*work_dir*⟩
./NanoSV -s /usr/bin/samtools -b ⟨*batchesfile*⟩ -o ⟨*output_file*⟩ ⟨*input_bam*⟩

2 The detailed data for SV callers result

The main manuscript shows the recall, precision, and F1 score of SVcnn and other SV callers in HG002, CHM13, and HG00733. The detailed statistics of SV number, recall, precision, and F1-score are shown in Tables S3 S4 S5 S6.

3 Generating benchmark lists of SVs

Although the Assemblytics result has high confidence, it is not perfect. It may miss some true SVs. To fill in the gap, we use HiFi reads to enhance the benchmarks further. For each sample (HG00733, HG002, or CHM13), we first align the HiFi reads on hg38 and obtain a sorted bam file; then, we run SVcnn and other four methods to obtain 5 lists of SVs. We observe that many SVs in these 5 lists are not included in the benchmark created by Assemblytics. To form a more comprehensive list of SVs, we merged the 5 lists of SVs together with Assemblytics' benchmark SV list and filtered the repeated SVs.

Although this combined list of SVs is comprehensive, it has a lot of noisy SVs. We validated every SV in the list using the sample genome as follows. Let G be the reference genome hg38. Let G' be the sample genome. Note that each SV replaces $G[st..ed]$ by a

Table S3: The number of insertions and deletions in the benchmark and called by different SV callers on the ONT datasets of HG002, CHM13, and HG00733.

methods	benchmark	SVcnn	cuteSV	Sniffles2	NanoSV(all)	DeBreak
HG002_DEL	9802	11206	12065	16472	11109	10018
HG002_INS	14005	15412	15726	19412	37906	13523
CHM13_DEL	6917	7728	10945	12354	16088	7118
CHM13_INS	10226	10686	12939	13704	28485	10264
HG00733_DEL	8960	10843	11946	16359	17390	9681
HG00733_INS	11750	15387	15841	17902	35670	13579

Table S4: The recall of the different SV callers on the ONT datasets of HG002, CHM13, and HG00733.

methods	SVcnn	DeBreak	cuteSV	Sniffles2	NanoSV(all)
HG002_DEL	89.5%	80.8%	89.3%	90.3%	55.2%
HG002_INS	89.7%	81.4%	86.4%	85.5%	85.7%
CHM13_DEL	89.5%	82.9%	91.1%	90.1%	89.5%
CHM13_INS	90.8%	83.2%	86.8%	85.7%	82.3%
HG00733_DEL	87.7%	77.6%	87.8%	88.7%	78.5%
HG00733_INS	91.6%	83.2%	89.3%	86.1%	86.7%

Table S5: The precision of the different SV callers on the ONT datasets of HG002, CHM13, and HG00733.

methods	SVcnn	DeBreak	cuteSV	Sniffles2	NanoSV(all)
HG002_DEL	78.2%	79.1%	72.5%	53.8%	48.7%
HG002_INS	81.6%	84.3%	76.9%	61.7%	31.7%
CHM13_DEL	80.1%	80.5%	57.2%	50.4%	38.2%
CHM13_INS	87.3%	83.2%	68.4%	64.2%	29.5%
HG00733_DEL	72.5%	71.8%	65.9%	48.6%	40.4%
HG00733_INS	69.9%	72.0%	66.2%	56.5%	28.6%

Table S6: The F1-score of the different SV callers on the ONT datasets of HG002, CHM13, and HG00733.

methods	SVcnn	DeBreak	cuteSV	Sniffles2	NanoSV(all)
HG002_DEL	0.83	0.80	0.80	0.67	0.52
HG002_INS	0.85	0.83	0.81	0.72	0.46
CHM13_DEL	0.85	0.82	0.70	0.65	0.54
CHM13_INS	0.89	0.83	0.77	0.73	0.43
HG00733_DEL	0.79	0.75	0.75	0.63	0.53
HG00733_INS	0.79	0.77	0.76	0.68	0.43

DNA segment R. (For deletion, R is an empty string. For insertion, $st = ed$. For inversion, R is the reverse complement of $G[st..ed]$.) Let S be the DNA segment $G[st - 10000..st - 1] \cdot R \cdot G[ed + 1..ed + 10000]$. We align S onto the sample genome G' using minimap2. We accept SV as correct if the following two criteria are true; otherwise, we filter the SV from the list.

- S aligns uniquely to G' with MAPQ $>$ 20 and the split part rate $<$ 10% (The split part rate is (front_split_length + behind_split_length)/S_length); and
- $G[st - 10000..st - 1]$ and $G[ed + 1..ed + 10000]$ is unique in G.

If G' is diploid, we align S onto the father genome and mother genome respectively. We accept SV as correct if there is one genome that meets the above two criteria. Through this method, we obtain a more complete and accurate benchmark.

4 The Venn diagram of new benchmark and GIAB benchmark

In order to test the reliability of generating a new benchmark method, we first use our method to get the new benchmark of HG002 on hg19. Next, we download the GIAB benchmark of HG002 on hg19. Then we compare our new benchmark with the GIAB benchmark and get the Venn diagram. From the Venn diagram (see Figure S1), we can find that our new benchmark contains almost 97% of SVs in the GIAB benchmark. In addition, our new benchmark also includes extra 11806 SVs (including 4694 DELs and 7112 INSs). Therefore, we conclude that the new benchmark is not only accurate but also more complete than the GIAB benchmark.

We also study the 411 SVs that are not included in our new benchmark. About 20% of the SVs may be false SVs, the specific examples have been shown in the Supplementary file Section 10. Except for these SVs, the most of remaining SVs are located in regions with poor mapping quality, so our new benchmark cannot judge whether these SVs are the true SVs. But this type of SVs only accounts for less than 2% of the GIAB benchmark, so it has little effect on the final results. Through the above method, we obtain three accurate and complete benchmarks.

5 Detect candidate SV by split read

For SVs whose two breakpoints are far, it is hard to detect them by CIGAR strings. Such SVs can be detected by split reads (i.e., reads that are aligned on two different loci on the reference genome). From the sorted BAM file, we retrain every read whose primary alignment is split and the MAPQ is at least 20 (by default). As shown in Figure S2, SVcnn detects the candidate SVs by the following rules.

(1) Suppose the read has two alignments and the two alignments are on the same chromosome and on the same strand. This split alignment probably contains a DEL or INS (see the top panel of Figure S2).

We label the two alignments as r1 and r2 (r1 is in front of r2). We record the two split points on the reference and label them as $loc1_{ref}$ (r1 split point) and $loc2_{ref}$ (r2 split point). Note that $loc2_{ref}$ may be in front of $loc1_{ref}$. The corresponding positions of the r1 split point and r2 split point on the read are denoted as $loc1_{read}$ and $loc2_{read}$. Next, we calculate the read_distance as $(loc2_{read} - loc1_{read})$ and the ref_distance as $(loc2_{ref} - loc1_{ref})$. If the read_distance minus the ref_distance is less than -200bp (by default), the split read is a candidate DEL. If the read_distance minus the ref_distance is greater than 200bp (by default), the split read is a candidate INS. The INS length is abs(read_distance−ref_distance). We still use a 6 tuples (chr_name, min($loc1_{ref}$, $loc2_{ref}$), max($loc1_{ref}$, $loc2_{ref}$), SV length, type, read_name) to record the candidate DEL or INS.

(2) Suppose the read has two alignments or three alignments and these alignments are on the same chromosome and have different strands. This split alignment probably contains an INV (see the bottom panel of Figure S2).

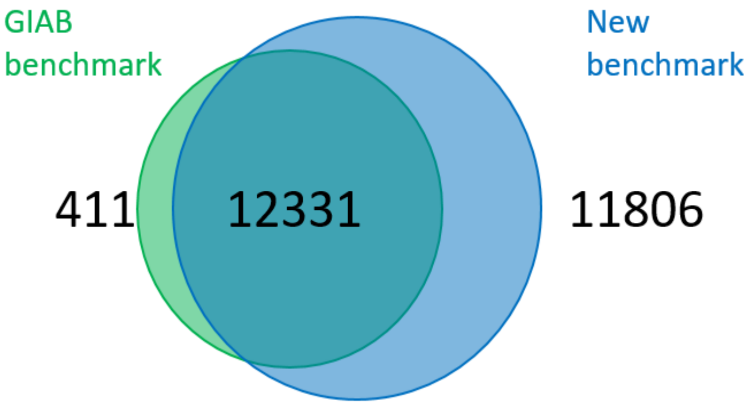

Figure S1: We compare our new benchmark with the GIAB benchmark and get this Venn diagram. From the Venn diagram, we can see that the new benchmark contains almost SVs in the GIAB benchmark, and only 411 SVs are not included in the new benchmark. In addition, the new benchmark also includes extra 11806 SVs.

If there are two alignments, we label the two alignments as r1 and r2 (r1 is in front of r2). We record the two split points' position in the reference and label them as $loc1_{ref}$ (r1 split point) and $loc2_{ref}$ (r2 split point). The SV length is $(loc2_{ref} - loc1_{ref})$. If the SV length exceeds 200bp (by default), the split read is denoted as a candidate INV. We use a 6 tuples (chr_name, $loc1_{ref}$, $loc2_{ref}$, SV length, INV, read_name) to record the candidate INV.

If there are three alignments, we label the three alignments as r1, r2, and r3 (r1 is in front of r2 while r2 is in front of r3). If the strand of r2 is different from that of r1 and r3, r2 may be a candidate INV. We record the two split points position of r1 and r3 in the reference and label them as $loc1_{ref}$ (r1 split point) and $loc3_{ref}$ (r3 split point). The SV length is $(loc3_{ref} - loc1_{ref})$. If the SV length exceeds 200bp (by default), the split alignment is a candidate INV. We use a 6 tuples (chr_name, $loc1_{ref}$, $loc3_{ref}$, SV length, INV, read_name) to record the candidate INV.

6 The detailed step to get the SVs in repeat regions

In the main manuscript, we observed that the vapor has a good performance in normal regions, but nor perform well in repeat regions. The repeat region of Hg19 and Hg38 are downloaded from the table browser (The website is http://genome.ucsc.edu/cgi-bin/hgTables). On the website, we download the Hg19 repeat regions by setting parameters just like the Figure S3.

Then we compare the SVs with the repeat regions. If over 80% part of an SV is in the repeat area, we consider that the SV is in the repeat regions.

7 Methods for checking whether SV caller output an SV in the benchmark

Different SV callers or benchmarks may report the same SV with slightly different positions and slightly different lengths. It is not easy to determine if two SVs are the same or not. Below, we describe a simple

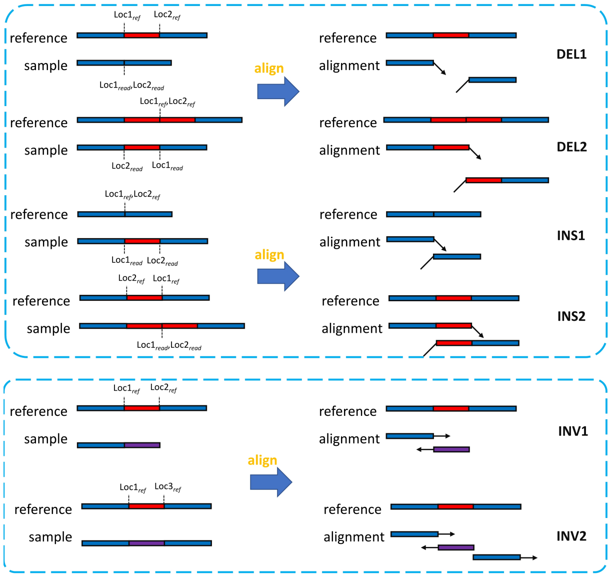

Figure S2: The left side of the figure shows the sample read and reference sequence for 6 types of SV, while the right side shows the alignment of the sample read on the reference. The top panel illustrates how to detect DEL and INS. For DEL, two examples are shown: One is the deletion of a non-repeat region and another one is the deletion of a tandem repeat. For INS, we also show two examples: One is the insertion in a non-repeat region while another one is a tandem duplication. The bottom panel illustrates how to detect the INV.

method to judge whether two SVs are the same SV.

7.1 Comparing two DELs

Consider two deletions DEL1: (start1_pos, end1_pos) and DEL2: (start2_pos, end2_pos). Set DEL1_len = end1_pos - start1_pos + 1 and DEL2_len = end2_pos - start2_pos + 1.

DEL1 and DEL2 are said to be the same if:

1. max(start_pos1,start_pos2)-min(end_pos1,end_pos2) less than 1000.

2. abs(DEL1_len-DEL2_len) <0.5*min(DEL1_len,DEL2_len).

7.2 Comparing two INSs

Consider two insertions INS1: (bp1, INS1_len) and INS2: (bp2, INS2_len)

INS1 and INS2 are said to be the same if:

1. abs(bp2-bp1) less than 1000.

2. abs(INS1_len-INS2_len) <0.5*min(INS1_len,INS2_len).

8 cluster reads with short SVs

For short SVs, sequencing noise has a great effect on the SV length. Hence, when two SVs are of similar length, it is hard to distinguish them as they are short. Here, we use hierarchical clustering to separate these reads. If we detect a bimodal distribution for all SV lengths, we report two heterozygous SVs. The detailed steps are as follows:

1. Filter 5% longest and shortest SV lengths.

2. Labeled every SV length as a cluster.

3. Repeat the following step if the number of clusters is more than 2.

   - Find two clusters with the smallest difference in cluster length and merge them into a new cluster. (The cluster length is the average of all SV lengths in the cluster.)

4. For the two remaining clusters. If the cluster length difference is less than 20+0.01*max(cluster1_length,cluster2_length), we merge them as one cluster.

For every cluster (cluster_type, cluster_length) obtained, we select an SV in the cluster that is the closest to the cluster_length to represent the cluster. These representative SVs will be used in the filtering step of the next section.

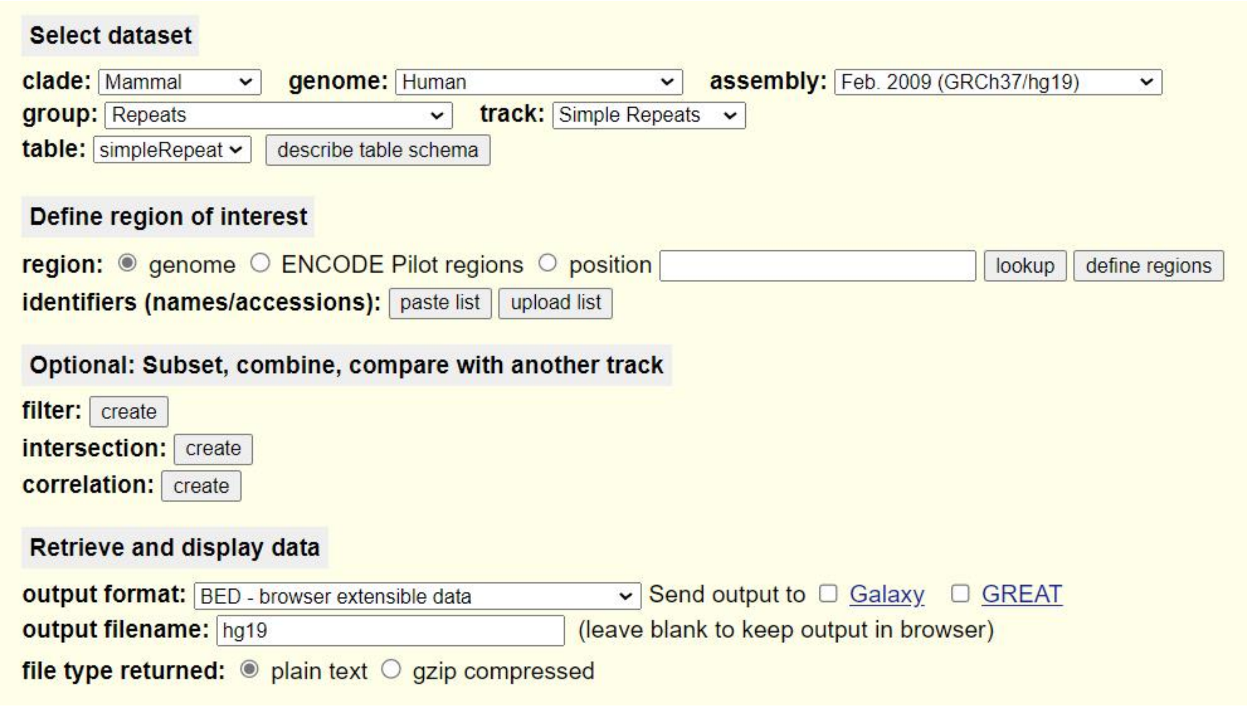

Figure S3: The parameters to get simple repeats are shown in the figure.

9 The detailed step to define simple repeat region in our detection method (SVcnn)

In the main manuscript, we find that long-read data have more noise in simple repeat regions (Especially regions like AAAAA or ATATAT). Hence, we check whether a candidate SV is in the repeat region and decide whether to filter it. For a region [start_pos, end_pos], if it meets one of the following criteria, we define this region as a simple repeat region. (region_len=end_pos-start_pos, A_num is the total number of A in this region, G_num is the total number of base G, C_num is the total number of base C, T_num is the total number of base T).

- A_num/region_len >0.9 or G_num/region_len >0.9 or C_num/region_len >0.9 or T_num/region_len >0.9.
- region has AAAAA and A_len (The length of AAAAA) is greater than 40% region_len (A can be replaced with G or C or T).
- There are two base_num/region_len >0.4 (The base_num is one of A_num, G_num, C_num and T_num).

The first criterion is to define the region AAAAAA. The second criterion is to define the region XXXXXXAAAAAA (XXXXXX is any sequence). The third criterion is to define the region ATATATATAT. After this step, we will further judge whether the candidate SV in this region is true or not.

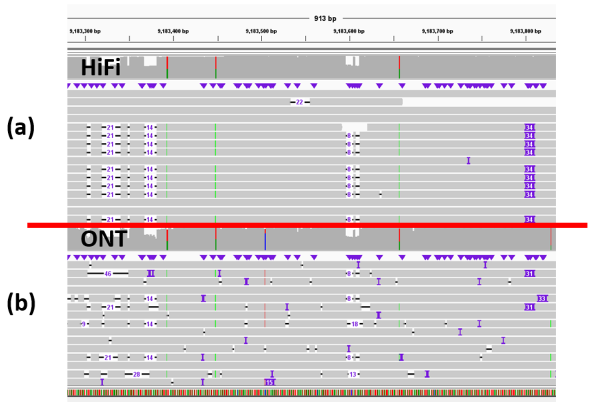

Figure S4: The IGV figure shows the region chr1:9183200-9183850 in hg19. According to the GIAB benchmark, there is an 84bp DEL in this region. (a) The top panel shows the IGV figure of the alignment of HiFi reads in this region. We can see that the HiFi reads only contain some small indels. (b) The bottom panel shows the IGV figure of the alignment of ONT reads on the same region. The ONT reads also only show some small indels. Hence, we judge that this SV is a false SV.

10 The false example in GIAB benchmark

We found dozens of SVs in the GIAB benchmark, which may be false SVs. Figure S4 shows a specific example. The GIAB benchmark shows that this region has a DEL with 84bp length (chr1:9183369-9183453 84bp). But whether it is on HiFi data or ONT data, the IGV figure of this region only shows a few small indels (less than 50bp). Hence, we think these SVs are false SVs that treat small indels as true SVs.

11 False SVs of current SV callers

This section aims to understand the reasons why current SV callers will miss some true SVs and output some false SVs. Figure S5 gives two examples.

The left panel in Figure S5 shows a region from HG002. When aligning HiFi data to the reference using NGMLR, we observe only one correct deletion (DEL) of 174bp in this region. However, due to noise and mismatches, most alignments of ONT data display two DELs, making it challenging to detect the correct DEL (174bp) using current methods. Moreover, these methods output one or two false DELs (57bp or 117bp). However, if we merge two DELs into one DEL (57+117) on the same read, we can still get a DEL with a length of about 174 bp. We note that such errors are mainly confined to repeat regions, as illustrated in Read4 of Figure 5 in the main manuscript, where one DEL may be incorrectly divided into multiple small DELs. To address this issue, we propose a solution for SV detection. Specifically, we first identify candidate SV regions and then merge the SVs on the same read within each region. Finally, we represent the merged SV as a single SV event. Our approach helps discover many true SVs and filters out many false SVs.

The right panel in Figure S5 shows a region from HG002. When aligning HiFi data to the reference using NGMLR, we can find that there is a pair of multi-allelic DELs (97bp and 62bp). While these two DELs are visible in the HiFi data, they are challenging to distinguish in the ONT data due to noise interference. Traditional SV callers rely on the difference in SV length to cluster SVs. However, when a region contains two SVs of the same type and similar length (multi-allelic SVs), noise interference makes it difficult to separate them using differences in SV length. Therefore, existing SV callers often report only one DEL. This challenge is more common for long-read data with high error rates but less frequent in HiFi data. To address this limitation, we utilize hierarchical clustering to differentiate long reads that contain multi-allelic SV pairs. This approach enables us to detect additional multi-allelic SVs, as described in

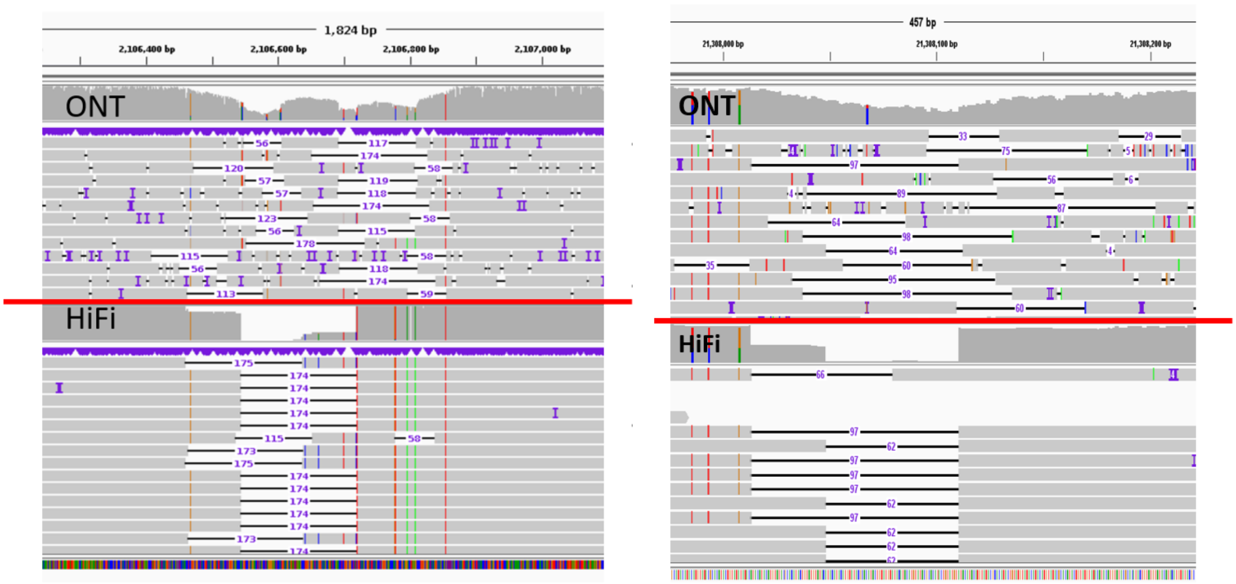

(a) (b)

Figure S5: This figure shows IGV graphs for two regions. The top panel shows the alignments (minimap2) of ONT data and the bottom panel shows the alignments (NGMLR) of HiFi data. In ONT data, the current SV callers have all obtained incorrect results in the three regions. **(a)** In the alignments of HiFi data, this region only has 174bp DEL. But most alignments in ONT data display two DELs. **(b)** In the alignments of HiFi data, this region has two DELs (97bp and 62bp). But it is difficult to distinguish these two DELs from ONT data. For figure (a), the current SV callers all output 57 bp DEL or 117 bp DEL in ONT data. For figure (b), the current SV callers all output only one DEL in ONT data.

detail in the result section.
